# Quantitative analysis of the impact of a human pathogenic mutation on the CCT5 chaperonin subunit using a proxy archaeal ortholog^☆^

**DOI:** 10.1016/j.bbrep.2017.07.011

**Published:** 2017-09-01

**Authors:** Dario Spigolon, D. Travis Gallagher, Adrian Velazquez-Campoy, Donatella Bulone, Jatin Narang, Pier Luigi San Biagio, Francesco Cappello, Alberto J.L. Macario, Everly Conway de Macario, Frank T. Robb

**Affiliations:** a Institute for Bioscience and Biotechnology Research (IBBR), Rockville, MD, USA; b Institute of Biophysics, UOS Palermo, National Research Council, Italy; c Department of Physics and Chemistry, University of Palermo, Palermo, Italy; d Institute of Biocomputation and Physics of Complex Systems (BIFI), Joint Units: BIFI-IQFR and GBsC-CSIC,Universidad de Zaragoza, Zaragoza, Spain; e Department of Biochemistry and Molecular and Cell Biology, Universidad de Zaragoza, Zaragoza, Spain; f Aragon Institute for Health Research (IIS Aragon), Zaragoza, Spain; g Fundacion ARAID, Government of Aragon, Zaragoza, Spain; h Department of Biomedicine and Clinical Neurosciences, Human Anatomy Section, University of Palermo, Palermo, Italy; i Euro-Mediterranean Institute of Science and Technology (IEMEST), Palermo, Italy; j Department of Microbiology and Immunology, School of Medicine, University of Maryland at Baltimore, Baltimore, USA; k Institute of Marine and Environmental Technology (IMET), Columbus Center, Baltimore, MD, USA

**Keywords:** Pf, *Pyrococcus furiosus*, Pf-CD1, *Pyrococcus furiosus* chaperonin subunit with the last 22 amino acids deleted, DSC, differential scanning calorimetry, ITC, isothermal titration calorimetry, DLS, dynamic light scattering, Chaperonopathies, CCT5, *Pyrococcus furiosus*, Chaperonin, Protein calorimetry, Neuropathy

## Abstract

The human chaperonin complex is a ~ 1 MDa nanomachine composed of two octameric rings formed from eight similar but non-identical subunits called CCT. Here, we are elucidating the mechanism of a heritable CCT5 subunit mutation that causes profound neuropathy in humans. In previous work, we introduced an equivalent mutation in an archaeal chaperonin that assembles into two octameric rings like in humans but in which all subunits are identical. We reported that the hexadecamer formed by the mutant subunit is unstable with impaired chaperoning functions. This study quantifies the loss of structural stability in the hexadecamer due to the pathogenic mutation, using differential scanning calorimetry (DSC) and isothermal titration calorimetry (ITC). The disassembly of the wild type complex, which is tightly coupled with subunit denaturation, was decoupled by the mutation without affecting the stability of individual subunits. Our results verify the effectiveness of the homo-hexadecameric archaeal chaperonin as a proxy to assess the impact of subtle defects in heterologous systems with mutations in a single subunit.

## Highlights

• A crippling hereditary neuropathy was addressed at the molecular level.
• The archaeal/CCT5 model represents a promising testbed for subtle defects.
• The homomeric archaeal model amplifies the effect of the mutation.
• The mutation decouples assembly without destabilizing individual subunits.

## Graphical abstract

**Figure:**
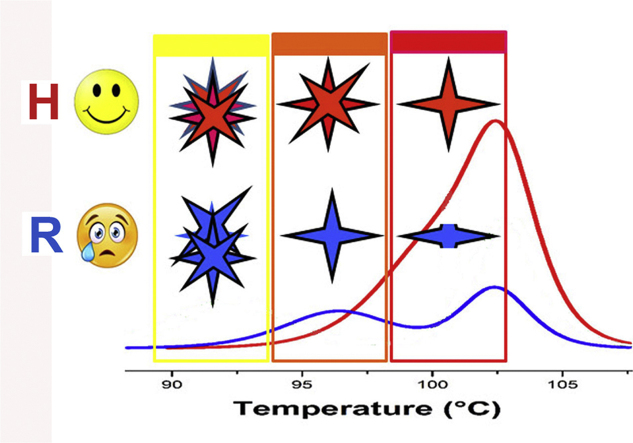

## 1 Introduction

Recent advances in human genomics are revealing numerous pathogenic germline and somatic mutations [1], also affecting chaperone genes [2]. However, elucidating mechanisms of functional deficits remains the bottleneck for pathogenetic analysis, which interferes with progress in understanding the mutations’ impact on protein homeostasis and therapeutics. Protein homeostasis is carried out mostly by the chaperoning system that, when faulty, leads to human disease. Here, we focus on the chaperonins, which are essential for protein folding and salvage pathways in all three Domains of life. The eukaryotic CCT (chaperonin containing TCP1), a Group II chaperonin, is an obligate chaperone for at least 10% of eukaryotic proteomes [3], [4]. Several CCT chaperonopathies are clinically characterized and information on their mode of inheritance exists [2], [5]. Group II chaperonins, e.g. CCT, are found in Archaea and eukaryotes, and these consist of two stacked rings of eight non-identical subunits per ring.

CCT is required for folding of various proteins such as actin, tubulin, and cell-cycle regulators eukaryotes [6], [7], [8], [9], [10], [11], and can suppress aggregation of toxic proteins [12], [13]. Defects in protein folding and other abnormalities, many associated with chaperonopathies, are typical of cancer, and chronic inflammatory, autoimmune and neurodegenerative disorders, [2], [5], [14], [15]. The CCT conformation induced by ATP hydrolysis is associated with substrate productive folding [16]. We modelled mutant forms of the CCT hexadecamer, using an archaeal ortholog with high sequence and structural homology to CCT5 [17]. We sought to elucidate the impact of a pathogenic mutation on the intrinsic properties and on the chaperoning functions of the human CCT5 subunit. The pathogenic mutation, His147Arg, is associated with a hereditary sensory neuropathy [18]. In this chaperonopathy, as in many others, understanding the molecular impact of pathogenic mutations is key to the design and development of specific chaperonotherapies [19].

Little is known about the impacts of pathogenic mutations on the intrinsic properties of chaperone molecules, and their functions. This is due to the multi-protein nature of the chaperoning machines, and to the scarcity of experimental models that would allow reproduction of the conditions seen in patients with chaperonopathies. We developed a model to study mutations in the CCT subunits and began to elucidate the impact of a pathogenic mutation on CCT5 structure and function [17], [20]. We investigated the mechanism of a sensory neuropathy caused by a point mutation (His147Arg) in CCT5, one of the eight subunits of the human CCT [18]. We introduced the pathogenic mutation into the ortholog chaperonin from a hyperthermophilic archaeon, *Pyrococcus furiosus* (Pf), which shares 44% sequence identity with the human CCT5 [17]. In the disease model, each of the 8 archaeal subunits in the two identical octamers that build the chaperoning hexadecamer carries the mutation. Therefore, the impact of the mutation is multiplied eight-fold per ring compared to the human CCT octamer. This amplification seems powerful for detecting subtle effects of a mutation, such as those which are likely to occur in humans, namely mutations that cause pathology but are compatible with survival. We found, previously, decreased stability and impaired chaperoning function of the mutant chaperonin [17]. Here, we used differential scanning calorimetry (DSC) and isothermal titration calorimetry (ITC), to quantitatively analyze the loss of structural stability in the hexadecamer containing the pathogenic mutation.

## 2 Materials and methods

### 2.1 Chaperonins

The molecules studied were Pf-CD1, Pf-CD1 Ile138His, and Pf-CD1 Ile138Arg, all derived from the *Pyrococcus furiosus* (Pf) chaperonin Group II Pf-Cpn [17; and Supplementary materials]. CD1 is a C-terminal deletion, produced by removing the last 22 residues of Pf-Cpn, a sequence segment that promotes solubility with minimal impact on function and stability. Pf-CD1 Ile138His represents the wild-type humanized version of the archaeal protein since it contains His at the site corresponding to the human mutation (Pf-H). Pf-CD1 Ile138Arg represents the pathogenic human mutant (Pf-R).

### 2.2 Protein production

The wild-type archaeal CCT subunit ortholog gene was amplified from *P. furiosus* genomic DNA, then modified and expressed in *Escherichia coli* to produce the three constructs as reported [17]. The pET33b(1) vector (Novagen, Madison, WI) was used for recombinant expression in *E. coli* BL21 (DE3). Expression details are given in Supplementary materials. Pure protein fractions were concentrated using 30 kDa spin filters (Amicon Milipore, Darmstadt, Germany) spun at 3500×*g* for 20 min. Protein homogeneity was assessed by running concentrated fractions on 12% SDS-PAGE gel and protein concentration was assessed using Bradford assay.

### 2.3 Differential scanning calorimetry (DSC)

Calorimetric experiments were conducted on a Nano-DSC (TA Instruments, New Castle, DE) with 0.3 mL capillary platinum cells. Details in Supplementary materials.

### 2.4 Isothermal titration calorimetry (ITC): oligomeric equilibrium

The protein oligomeric equilibrium was studied using a Nano ITC Low Volume (TA Instruments) with a reaction cell volume of 1 mL kept at 25 °C. The procedure was recently described [21], [22], [23]. Details in Supplementary materials.

### 2.5 Isothermal titration calorimetry: ATP binding

ATP binding was studied with a Nano ITC Low Volume (TA Instruments). Details in Supplementary materials.

## 3 Results

**Fig. 1:**
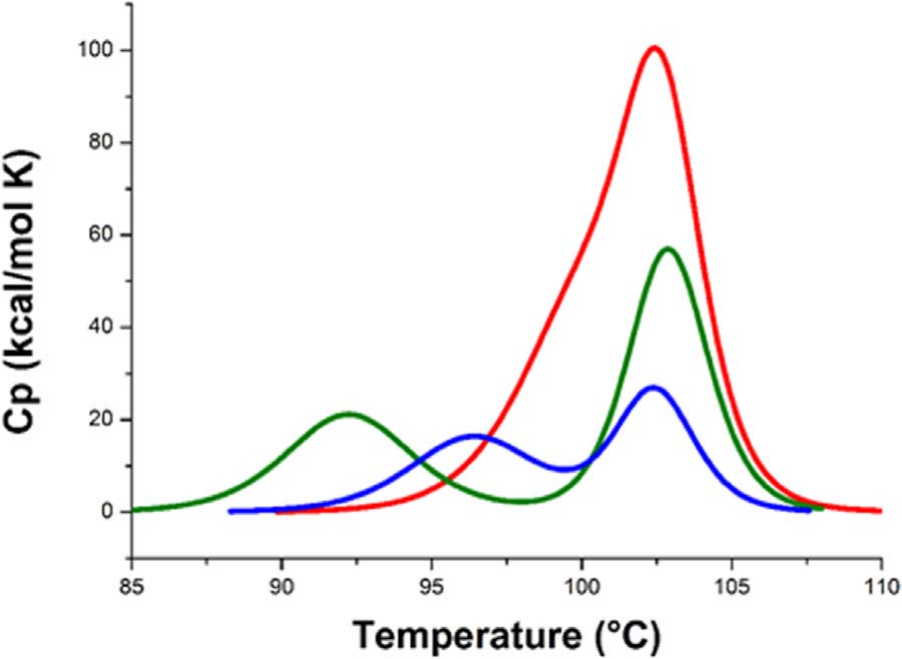
DSC. Thermograms for Pf-CD1 (red), Pf-H (green), and Pf-R (blue), all at 7 μM. The proteins show biphasic melting, but in Pf-CD1 the two peaks overlap, implying closer coupling between hexadecamer disassembly and denaturation of monomers. Calorimetric traces are given after subtraction of the instrumental base line. The scan rate was 60 °C/h.

We compared the thermal unfolding of Pf-CD1, Pf-H and Pf-R under the same conditions (scan rate, 60 °C/h, and protein concentration, 0.3 mg/mL), Fig. 1. The results showed a considerable difference in structural stability between the proteins, as demonstrated by the strong difference in calorimetric enthalpy, revealing a different energetic ability to maintain the complex structure, particularly for the pathogenic mutant Pf-R (182 kcal/mol, versus 308 and 515 kcal/mol, for Pf-H and Pf-CD1, respectively, Table 1).

**Table 1:** Thermodynamic parameters for heat denaturation and effect of nucleotide binding.

Protein^a^	*Tm* (°C)	*∆H*_*cal*_^b^ (kcal/mol)	*∆H*_*vh*_ (kcal/mol)	*R*^c^
Pf-CD1	102.6	515	214.2	2.4
Pf-H	103.2	308	197.2	1.56
Pf-R	102.7	182	160.3	1.14
Pf-CD1-Mg-ATP	102.5	365	239	1.52
Pf-H-Mg-ATP	99.6	518	156	3.3
Pf-R-Mg-ATP	99.7	157	200	0.8

a 7 μM.

b *∆H*_*cal*_ and *∆H*_*vh*_per subunit.

c R = ∆H_cal_/∆H_vh_.

**Fig. 2:**
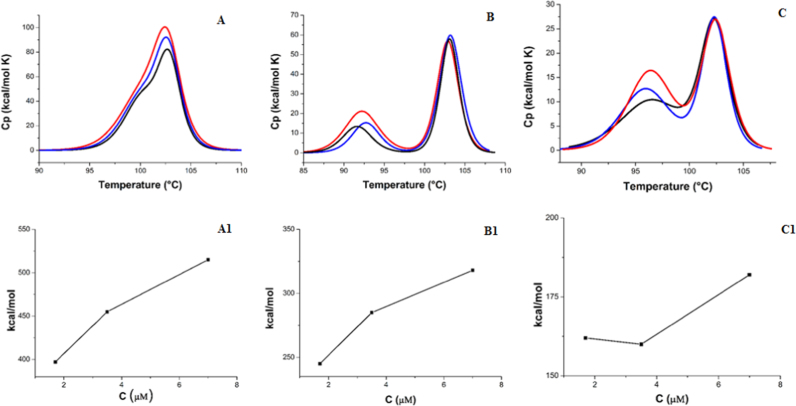
DSC at different protein concentrations: 7 μM (red), 3.5 μM (blue), and 1.7 μM (black). **A**, **B**, **C**, thermograms for Pf-CD1, Pf-H, and Pf-R, respectively. **A1**, **B1**, and **C1**, variations in total measured enthalpy as a function of concentration.

Pf-CD1 showed more stability with respect to Pf-H and Pf-R and the highest ability to maintain the complex structure. The asymmetry observed in the DSC thermograms is evident for all proteins (particularly for Pf-H and Pf-R), and all peaks are skewed at temperatures below the transition midpoint (T_m_), where the transition is less sharp and deviates more from the two-state fit, as expected for a transition coupled to dissociation [21], [25]. Consistent with a transition coupled to dissociation, the calorimetric enthalpy increased with protein concentration, Fig. 2. These results indicate that the unfolding of Pf-CD1, Pf-H, and Pf-R is coupled with the dissociation of the oligomers, showing a different tendency to dissociate for the three proteins at increasing temperatures.

ITC enabled assessment of the oligomeric equilibrium of the three chaperonins, by revealing the fractions of monomers and oligomers as a function of concentration. The presence of monomers at high protein concentration, especially for Pf-R, was confirmed by native 4–9% gradient native-PAGE analysis [17]. The oligomers' dissociation constants *K*_*d*_, along with *∆H*_*d*_ and other thermodynamic parameters calculated for the oligomeric equilibria of Pf-CD1, Pf-H, and Pf-R are in Table 2.

**Table 2:** Thermodynamic parameters for the hexadecamer-monomer equilibria.^a^

Protein	*k*_*d*_ (μM)	*∆G*_*d*_ (kcal/mol)	*∆S*_*d*_ (kcal/mol K)	*∆H*_*d*_ (kcal/mol)
Pf-CD1	0.31 ± 0.06	− 8.8 ± 0.1	− 1.14 ± 0.01	− 350 ± 5
Pf-H	0.43 ± 0.05	− 8.7 ± 0.24	− 0.81 ± 0.02	− 250 ± 7
Pf-R	0.1 ± 0.05	− 9.5 ± 0.3	− 0.13 ± 0.04	− 49 ± 1.7

a All parameters are expressed per subunit.

**Fig. 3:**
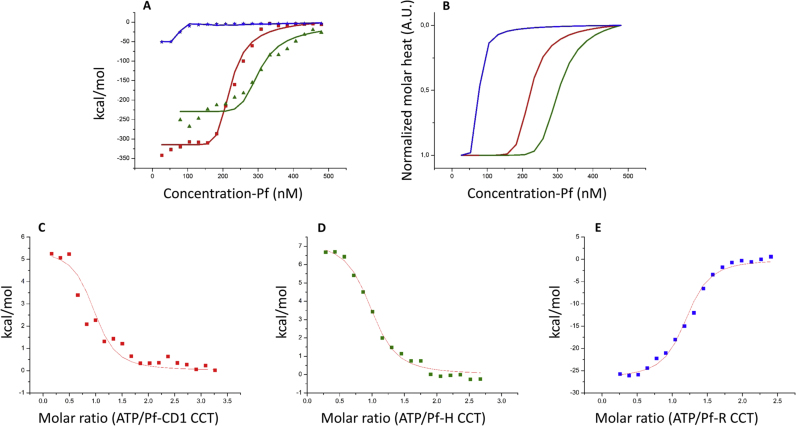
ITC. **A** and **B**, ITC of the oligomer assembly process for Pf­ CD 1 (red), Pf-H (green), and Pf-R (blue). **A**, measured heat for 18 successive injections and best fit to a two-state curve according to a hexadecamer-monomer equilibrium. **B**, the corresponding normalized molar heat functions. **C**-**E**, ITC of ATP binding to the chaperonins. **C** (Pf-CDl), **D** (Pf-H), and **E** (Pf-R), enthalpies as a function of ATP stoichiometry, with the best-fit curves assuming a single binding site per subunit. A molar ratio of 16 corresponds to one ATP per subunit.

The oligomeric equilibrium of the three chaperonins was observed by ITC, Fig. 3, A and B. The monomer fraction and the total monomer concentration in the ITC reaction cell for hexadecamer-monomer equilibrium are related through a sixteenth-order polynomial equation (see Section 2, and Table 2 for thermodynamic parameters). The data from the ITC dilution experiments show that Pf-CD1, Pf-H, and Pf-R disassembly involves a favorable and considerable enthalpy (negative) change. Therefore, the assembly of the hexadecameric complex is endothermic and is entropically driven. Moreover, the negative Gibbs free energy indicates that, for all the three proteins, disassembly is a thermodynamically favorable reaction as long as the concentration is less than the critical transition concentration (CTC). Thus, protein dilution promotes disassembly and the increase in concentration favors assembly. The hexadecamer-monomer model superimposes well on the experimental data, indicating that no intermediate states are involved.

ATP binding was also investigated by ITC, Fig. 3C–E. Pf-CD1, Pf-H, and Pf-R were shown to bind ATP. The three proteins showed different ATP-binding mechanisms, particularly Pf-R had an exothermic binding enthalpy *∆H* (− 26.8 kcal/mol) compared to the endothermic transitions of Pf-CD1 and Pf-H that were 4.3 and 6.5 kcal/mol, respectively, Table 3. Our results indicate that only the nucleotide endothermic binding to Pf-H and Pf-CD1 leads to conformational changes induced by Mg-ATP.

**Table 3:** ATP-binding parameters obtained by the “one set of sites” model.^a^

**Protein**	***n***	***k***_***d***_**(μM)**	***∆S*****(cal/mol K)**	***∆H*****(kcal/mol)**
Pf-CD1	0.93 ± 0.15	0.32 ± 0.05	47.3 ± 5.5	5 ± 1.5
Pf-H	0.95 ± 0.06	0.3 ± 0.02	53.2 ± 5.8	7 ± 0.7
Pf-R	1.16 ± 0.05	0.2 ±0.01	− 55.4 ±3.1	− 26.7 ± 1.3

a All parameters are expressed per monomer.

**Fig. 4:**
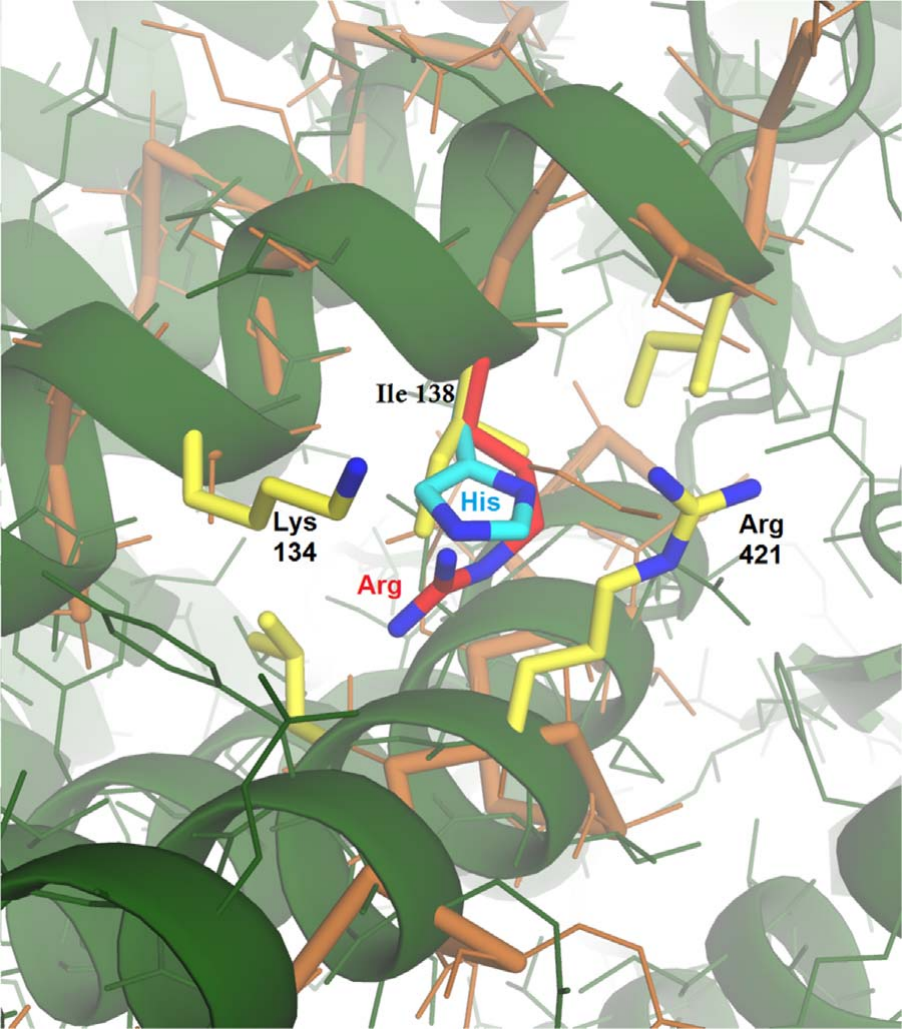
Protein structure around the mutation site, showing the superposed crystal structures from *Thermococcus* (Tk, PDB:1Q3Q, green) and yeast (4V81, orange). The *Thermocuccus* and Pf sequences are >90% identical, so the green structure gives a reliable model for Pf. The yeast structure overlays closely, consistent with the high homology of all known CCT sequences, and enabling 4V81 here to represent the human structure. The mutation site (Ile-138 in both archaea, yellow) and its close neighbors, which are identical in Tk and Pf. The mutation site is just below the protein surface in a sterically restricted pocket in all the homologs. In human CCT5, the wild-type residue corresponding to Ile-138 is a His (cyan). The pathological mutation replaces His with Arg (red). Although His is accommodated in the normal human protein, the Arg side chain appears to be sterically and electrostatically unfavorable in both the archaeal and human frameworks. Image made using PyMOL Molecular Graphics System, Version 1.5, Schrodinger.

We also confirmed this finding by examining the ATP binding effect using circular dichroism [24]. We found that the R mutant (Pf-R) is energetically less efficient at maintaining the oligomeric complex (Fig. S1, and Table S1), as indicated also by the variation in the calorimetric enthalpy and by its elution profile in SE-HPLC. Thus the mutation appears to affect the oligomer's thermodynamic stability and its ability for effective self-organization in vitro. This is consistent with simple structure-based predictions because of the relative sizes Ile<His<Arg as shown by molecular modeling, Fig. 4. The space for the substituted side chain is tightly constrained by neighboring residues. Moreover, the side chains’ net ionic charge follows the same order as that of residues sizes, strengthening the structure- based predictions of destabilization.

## 4 Discussion

Thermodynamic analysis of oligomeric proteins provides insight into their self-organization and their response to stressors and to mutations. For heterologous proteins, such as the CCT complex, which has contributions from eight paralogous subunits, the stability of the protein may be subtly compromised by a point mutation such as the His147Arg occurring in only one subunit. Our study is novel in that we are studying a homohexadecamer, amplifying the effect of the mutation eightfold per ring, Fig. S2. The CCT5 subunit has been successfully expressed as a homooligomeric complex [26] in both wild type as well as the His147Arg allele [27], and was found to have a subtle defect in chaperoning ability [27]. Here, we have examined the effects of the His147Arg mutation in an archaeal system that allowed the disassembly and ATP binding to be examined by DSC, ITC, and gel permeation.

The oligomer's thermal denaturation appeared as a multistep process and the ratio between calorimetric enthalpy and van’t Hoff enthalpy per subunit suggested a non-2-state condition. The three proteins showed differences in stability, especially in calorimetric enthalpy, revealing different energetic capacities for maintaining the complex structure, particularly for Pf-R (182 kcal/mol) versus Pf-H (308 kcal/mol) and Pf-CD1 (515 kcal/mol). The Pf-CD1 unfolding transition was observed at higher temperature, indicating its greater capacity to maintain the hexadecamer structure. Moreover, the asymmetry observed in the DSC thermograms is evident for all proteins (particularly for Pf-H, and Pf-R), and all peaks are skewed at temperatures below the transition midpoint, where the transition is less sharp and deviates more from the two-state fit, as expected for a transition coupled to dissociation [23]. These results indicate that the dissociation of all three proteins is coupled with unfolding, but to a different extent. The multistep process is also consistent with the observed ratios greater than 1.0, a characteristic value for a two state thermal denaturing transition [28], of the calorimetrically determined enthalpies (∆*H*_*cal*_) to the corresponding van't Hoff enthalpies (∆*H*_*vh*_) per subunit.

Further support for the multistep-disassembly process was obtained by carrying out measurements at various concentrations at the same scan rate, showing that the calorimetric enthalpy generally increased with protein concentration as expected for a transition coupled to dissociation.

The overall enthalpy change may include three main contributing factors: (*i*) Interaction enthalpy between subunits (*∆H*_*i*_); (*ii*) Conformational enthalpy of subunits (*∆H*_*c*_); and (*iii*) Solvation enthalpy of subunits (*∆H*_*s*_) [29]. *∆H*_*i*_ corresponds to the enthalpy changes from non-covalent interactions such as hydrogen bonds, electrostatic forces, and van der Waals interactions. *∆H*_*s*_ represents the enthalpy due to the uptake or release of the ordered water molecules from the contact interface (hydration-dehydration phenomena); the desolvation of polar and nonpolar groups is enthalpically unfavorable (although more unfavorable for polar groups). *∆H*_*c*_ is related to the conformational enthalpy when an ordered secondary/tertiary/quaternary structure of the protein is formed. Considering the unfavorable positive enthalpy value of Pf-CD1 assembly, we conclude that *∆H*_*c*_ *+ ∆H*_*i*_ values are exothermic. Oligomer dissociation with negative enthalpy also implies that the assembly process is endothermic and entropically driven [30]. Therefore, the total endothermic enthalpy of monomer binding is attributed to significant unfavorable and, thus, positive solvation enthalpy due mostly to the release of ordered water molecules from the monomer-oligomer interface. Protein folding and ligand binding to proteins through hydrophobic interactions are often accompanied by the burial of nonpolar surfaces from water [31]. The positive entropy associated with Pf-proteins assembly follows the classical hydrophobic effect, which is an entropy-driven process. Partitioning of a nonpolar molecule from water to a nonpolar phase is accompanied by an increase in the entropy of the system. Since the Pf-protein interfaces are mostly hydrophobic, there may be a large number of ordered waters released upon assembly. Thus, both the favorable entropy and the unfavorable enthalpy can be explained by a release of ordered water molecules upon hexadecamer assembly, especially in the case of Pf-CD1, where the hydrophobicity is higher than for Pf-H and Pf-R. *∆H*_*c*_ may additionally contribute to the endothermic overall effect.

ATP binding was investigated by ITC. Pf-CD1, Pf-H, and Pf-R were shown to bind ATP, but apparently with different mechanisms, Fig. S3. The binding isotherms were analyzed with a model considering several identical and independent binding sites. In this model, the chaperonin has 16 identical nucleotide binding sites, which are considered to be independent of each other with a uniform binding constant, *K*_*a*_, and enthalpy change, *∆H*. The best fitting parameter values are listed in Table 3. The theoretical curves showed a good match with the experimental data for ATP. The three proteins showed different ATP-binding mechanisms: in particular Pf-R shows an exothermic binding enthalpy *∆H* (− 26.8 kcal/mol) compared to the endothermic ones of Pf-CD and Pf-H that were 4.3 and 6.5 kcal/mol, respectively.

Our results indicate that the nucleotide endothermic binding to Pf-H, and to Pf-CD1, leads to conformational changes induced by Mg-ATP, showing the transition of the complex to the closed conformation, which is usually required for ATP hydrolysis to bring the lid helices into close proximity during the conformational cycling. Conformational changes in CCT have also been observed by electron microscopy [32]. Opening of the lid occurs in conjunction with releasing ADP from the active site. The complex can exist in an asymmetrical conformation with one ring closed and one open, even during ATP cycling conditions, suggesting an inter-ring allosteric model mediated through a two-stroke mechanism [33]. However, the allosteric communication that occurs between the rings is not well understood.

CD spectra of the three proteins in their native and denatured thermal state were recorded at 190–250 nm to observe the effect of nucleotide binding on the secondary structure (Fig. S1 and Table S1). The results showed that the three chaperonin variants undergo conformational changes upon ATP binding. Pf-CD1 and Pf-H became more structured upon ATP binding, whereas Pf-R lost structure and resembled the denatured state to some degree.

In conclusion, the three chaperonin molecules tested have distinct thermodynamic profiles, showing different disassembly behavior and implying that there are different cooperativity levels and different conformational trajectories. The pathogenic R mutant (Pf-R) is energetically the weakest at maintaining the oligomeric complex and displays a distinct oligomeric equilibrium characterized by the lowest enthalpy of dissociation (∆*H*_*d*_). In a previous study we noted that the presence of the Arg residue replacing Ile was likely sterically unfavorable based on structural modeling [17]. The His residue apparently has a less severe effect on complex stability than Arg as expected for a less bulky sidechain. Thus, the pathogenic mutation appears to reduce the hexadecamer's stability and capacity for reassembly to form a stable complex. The observed differences in ATP binding provide further evidence that the R mutation in Pf-R destabilizes the chaperonin's structure, Fig. 4, and thereby also interrupts the allosteric cycle by failing to induce the normal conformational changes in neighboring subunits. Our study is enabling an approach that leads to the amplification of a subtle effect, which arguably represents the majority of mutations in viable human carriers, since profound dysfunction in one subunit of these key complexes would be lethal. Information obtained with this natural system can very well help expand our knowledge on the impact of a mutation on chaperonin subunits alongside what can be learned by using the human CCT5 molecule and its oligomers in vitro [27], [28]. Both systems can be considered complementary rather than mutually exclusive.

## Funding

Supported partially by Italian MIUR grant PON02_00355_2964193 and by PO. FESR 2007/2013 “Piattaforma regionale di ricerca traslazionale per la salute” 4.1.2. Asse IV. AJLM, FC, and ECdeM were partially supported by the Euro-Mediterranean Institute of Science and Technology, Italy.

## Authors contribution

D.S., performed DSC and ITC experiments, contributed to data analysis, and wrote the manuscript; J.N., implemented protein expression; D.T.G. constructed molecular models and performed data analysis; A.V.C., developed models for ITC data analysis and interpreted DSC and ITC results; D.B., conducted CD experiments, analyzed data, and contributed to data interpretation; P.L.S.B. and F.C., contributed to implementation and coordination of the project, data analysis, and edited the manuscript; A.J.L.M, E.C. de M., and F.T.R., conceived the research; guided the experiments, interpreted the data, and wrote the manuscript.

## Footnotes

Appendix A Supplementary data associated with this article can be found in the online version at doi:10.1016/j.bbrep.2017.07.011.

## References

[1] Shendure J., Akey J.M. (2015). The origins, determinants, and consequences of human mutations. Science.

[2] Macario A.J.L., Conway de Macario E. (2005). Sick chaperones, cellular stress and disease. N. Engl. J. Med..

[3] Joachimiak J.A., Walzthoeni T., Liu Corey W., Aebersold R., Frydman J. (2014). The structural basis of substrate recognition by the eukaryotic chaperonin TRiC/CCT. Cell.

[4] Lopez T., Dalton K., Frydman J. (2015). The mechanism and function of Group II chaperonins. J. Mol. Biol..

[5] Macario A.J.L., Conway de Macario E., Cappello F. (2013). The Chaperonopathies. Diseases with Defective Molecular Chaperones.

[6] Rommelaere H., Van Troys M., Gao Y., Melki R., Cowan N.J., Vandekerckhove J., Ampe C. (1993). Eukaryotic cytosolic chaperonin contains t-complex polypeptide 1 and seven related subunits. Proc. Natl. Acad. Sci. USA.

[7] Chen X., Sullivan D.S., Huffaker T.C. (1994). Two yeast genes with similarity to TCP-1 are required for microtubule and actin function in vivo. Proc. Natl. Acad. Sci. USA.

[8] Thulasiraman V., Yang C.F., Frydman J J. (1999). In vivo newly translated polypeptides are sequestered in a protected folding environment. EMBO J..

[9] Spiess C., Meyer A.S., Reissmann S., Frydman J. (2004). Mechanism of the eukaryotic chaperonin: protein folding in the chamber of secrets. Trends Cell Biol..

[10] Yam A.Y., Xia Y., Lin H.T., Burlingame A., Gerstein M., Frydman J. (2008). Defining the TRiC/CCT interactome links chaperonin function to stabilization of newly made proteins with complex topologies. Nat. Struct. Mol. Biol..

[11] Amit M., Weisberg S.J., Nadler-Holly M., McCormack E.A., Feldmesser E., Kaganovich D., Willison K.R., Horovitz A. (2010). Equivalent mutations in the eight subunits of the chaperonin CCT produce dramatically different cellular and gene expression phenotypes. J. Mol. Biol..

[12] Kitamura A., Kubota H., Pack C.G., Matsumoto G., Hirayama S., Takahashi Y., Kimura H., Kinjo M., Morimoto R.I., Nagata K. (2006). Cytosolic chaperonin prevents polyglutamine toxicity with altering the aggregation state. Nat. Cell Biol..

[13] Shahmoradian S.H., Galaz-Montoya J.G., Schmid M.F., Cong Y., Ma B., Spiess C., Frydman J., Ludtke S.J., Chiu W. (2013). TRiC's tricks inhibit huntingtin aggregation. eLife.

[14] Gregersen N., Bross P., Jorgensen M.M., Corydon T.J., Andresen B.S. (2000). Defective folding and rapid degradation of mutant proteins is a common disease mechanism in genetic disorders. J. Inherit. Metab. Dis..

[15] Dobson C.M. (2004). Principles of protein folding, misfolding and aggregation. Semin. Cell Dev. Biol..

[16] Meyer A.S., Gillespie J.R., Walther D., Millet I.S., Doniach S., Frydman J. (2003). Closing the folding chamber of the eukaryotic chaperonin requires the transition state of ATP hydrolysis. Cell.

[17] Min W., Angileri F., Luo H., Lauria A., Shanmugasundaram M., Almerico A.M., Cappello F., Conway de Macario E., Lednev I.K., Macario A.J.L., Robb F.T. (2014). A human CCT5 gene mutation causing distal neuropathy impairs hexadecamer assembly in an archaeal model. Sci. Rep..

[18] Bouhouche A., Benomar A., Bouslam N., Chkili T., Yahyaoui M. (2006). Mutation in the epsilon subunit of the cytosolic chaperonin-containing t-complex peptide-1 (Cct5) gene causes autosomal recessive mutilating sensory neuropathy with spastic paraplegia. J. Med. Genet..

[19] Cappello F., Marino Gammazza A., Palumbo Piccionello A., Campanella C., Pace A., Conway de Macario E., Macario A.J.L. (2014). Hsp60 chaperonopathies and chaperonotherapy: targets and agents. Expert Opin. Ther. Targets.

[20] Luo H., Laksanalamai P., Robb F.T. (2009). An exceptionally stable Group II chaperonin from the hyperthermophile *Pyrococcus furiosus*. Arch. Biochem. Biophys..

[21] Freire E. (1989). Statistical thermodynamic analysis of the heat capacity function associated with protein folding-unfolding transitions. Commun. Mol. Cell. Biophys..

[22] Luke K., Apiyo D., Wittung-Stafshede P. (2005). Dissecting homo-heptamer thermodynamics by isothermal titration calorimetry: entropy-driven assembly of co-chaperonin protein 10. Biophys. J..

[23] Barranco-Medina S., Kakorin S., Lázaro J.J., Dietz K.J. (2008). Thermodynamics of the dimer-decamer transition of reduced human and plant 2-cys peroxiredoxin. Biochemistry.

[24] Sreerama N., Woody R.W. (2000). Estimation of protein secondary structure from circular dichroism spectra: comparison of CONTIN, SELCON, and CDSSTR methods with an expanded reference set. Anal. Biochem..

[25] Cohen S.S., Riven I., Cortajarena A.L., De Rosa L., D'Andrea L.D., Regan L., Haran G. (2015). Probing the molecular origin of native-state flexibility in repeat proteins. J. Am. Chem. Soc..

[26] Sergeeva O.A., Chen B., Haase-Pettingell C., Ludtke S.J., Chiu W., King J.A. (2013). Human CCT4 and CCT5 chaperonin subunits expressed in *Escherichia coli* form biologically active homo-oligomers. J. Biol. Chem..

[27] Sergeeva O.A., Tran M.T., Haase-Pettingell C., King J.A. (2014). Biochemical characterization of mutants in chaperonin proteins CCT4 and CCT5 associated with hereditary sensory neuropathy. J. Biol. Chem..

[28] Privalov P.L., Dragan A.I. (2007). Microcalorimetry of biological macromolecules. Biophys. Chem..

[29] Lakshminarayanan R., Fan D., Du C., Moradian-Oldak J. (2007). The role of secondary structure in the entropically driven amelogenin self-assembly. Biophys. J..

[30] Abraham T., Lewis R.N., Hodges R.S., McElhaney R.N. (2005). Isothermal titration calorimetry studies of the binding of a rationally designed analogue of the antimicrobial peptide gramicidin S to phospholipid bilayer membranes. Biochemistry.

[31] Barisas B.G., Gill S.J. (1978). Microcalorimetry of biological systems. Ann. Rev. Phys. Chem..

[32] Cong Y., Schroder F.G., Meyer A.S., Jakana J., Ma B., Dougherty M.T., Schmid M.F., Reissmann S., Levitt M., Ludtke S.L., Frydman J., Chiu W. (2011). Symmetry-free cryo-EM structures of the chaperonin TRiC along its ATPase-driven conformational cycle. EMBO J..

[33] Reissmann S., Parnot C., Booth C.R., Chiu W., Frydman J. (2007). Essential function of the built-in lid in the allosteric regulation of eukaryotic and archaeal chaperonins. Nat. Struct. Mol. Biol..

