# Supplementary material for: Quantitative analysis of the impact of a human pathogenic mutation on the CCT5 chaperonin subunit using a proxy archaeal ortholog
Source: Biochem Biophys Rep. 2017 Sep 1;12:66–71. doi: 10.1016/j.bbrep.2017.07.011 (PMC5851525; doi:10.1016/j.bbrep.2017.07.011)
Supplement: Supplementary file 2 — Supplementary material [file mmc2.docx]

**Supplementary Materials**

**Amino-acid sequences**

***Pyrococcus furiosus* (archaeal) CCT subunit: FASTA sequences**

***Pyrococcus furiosus* chaperonin (the archaeal Pf-Cpn). Ile (I) at position 138.**

**>gi|18978346|ref|NP_579703.1| thermosome, single subunit [*Pyrococcus furiosus* DSM 3638].**

**Name: Pf-Cpn (549 amino acids). Note: this molecule was not studied in the work presented but it is shown here because it is the master molecule from which all studied mutants are derived.**

MAQLAGQPILILPEGTQRYVGRDAQRMNILAARIVAETIRTTLGPKGMDKMLVDSLGDIVITNDGATILD

EMDIQHPAAKMMVEVAKTQDKEAGDGTTTAVVIAGELLRKAEELLDQNIHPSIIIKGYTLAAQKAQEILE

NIAKEVKPDDEEILLKAAMTSITGKAAEEEREYLAKLAVEAVKLVAEKEDGKYKVDIDNIKLEKKEGGSV

RDTQLIRGVVIDKEVVHPGMPKRVEKAKIALINDALEVKETETDAEIRITSPEQLQAFLEQEERMLREMV

EKIKEVGANVVFVQKGIDDLAQHYLAKYGIMAVRRVKKSDMEKLAKATGAKIVTNIRDLTPEDLGYAELV

EERKVAGESMIFVEGCQNPKAVTILIRGGTEHVVDEVERALEDAIKVVKDILEDGKILAGGGAPEIELAI

RLDEYAKEVGGKEQLAIEAFAEALKVIPRTLAENAGLDPIETLVKVIAAHKEKGPTIGVDVYEGEPADML

ERGVIEPLRVKKQAIKSASEAAIMILRIDDVIAASKL**EKEKEKEGEKGGGSEDFSSDLD**

**Mutants studied**

***Pyrococcus furiosus* chaperonin Pf-Cpn mutant CD1, namely the wild type without the last 22 amino acids**

**Name: Pf-CD1. (527 amino acids).**

MAQLAGQPILILPEGTQRYVGRDAQRMNILAARIVAETIRTTLGPKGMDKMLVDSLGDIVITNDGATILD

EMDIQHPAAKMMVEVAKTQDKEAGDGTTTAVVIAGELLRKAEELLDQNIHPSIIIKGYTLAAQKAQEILE

NIAKEVKPDDEEILLKAAMTSITGKAAEEEREYLAKLAVEAVKLVAEKEDGKYKVDIDNIKLEKKEGGSV

RDTQLIRGVVIDKEVVHPGMPKRVEKAKIALINDALEVKETETDAEIRITSPEQLQAFLEQEERMLREMV

EKIKEVGANVVFVQKGIDDLAQHYLAKYGIMAVRRVKKSDMEKLAKATGAKIVTNIRDLTPEDLGYAELV

EERKVAGESMIFVEGCQNPKAVTILIRGGTEHVVDEVERALEDAIKVVKDILEDGKILAGGGAPEIELAI

RLDEYAKEVGGKEQLAIEAFAEALKVIPRTLAENAGLDPIETLVKVIAAHKEKGPTIGVDVYEGEPADML

ERGVIEPLRVKKQAIKSASEAAIMILRIDDVIAASKL

***Pyrococcus furiosus* chaperonin Pf-Cpn mutant CD1, namely the wild type without the last 22 amino acids, with His (H) instead of Ile at position 138 (Ile138His).**

**Name: Pf-CD1 His 138; Pf-H in short. (527 amino acids).**

MAQLAGQPILILPEGTQRYVGRDAQRMNILAARIVAETIRTTLGPKGMDKMLVDSLGDIVITNDGATILD

EMDIQHPAAKMMVEVAKTQDKEAGDGTTTAVVIAGELLRKAEELLDQNIHPSIIIKGYTLAAQKAQEHLE

NIAKEVKPDDEEILLKAAMTSITGKAAEEEREYLAKLAVEAVKLVAEKEDGKYKVDIDNIKLEKKEGGSV

RDTQLIRGVVIDKEVVHPGMPKRVEKAKIALINDALEVKETETDAEIRITSPEQLQAFLEQEERMLREMV

EKIKEVGANVVFVQKGIDDLAQHYLAKYGIMAVRRVKKSDMEKLAKATGAKIVTNIRDLTPEDLGYAELV

EERKVAGESMIFVEGCQNPKAVTILIRGGTEHVVDEVERALEDAIKVVKDILEDGKILAGGGAPEIELAI

RLDEYAKEVGGKEQLAIEAFAEALKVIPRTLAENAGLDPIETLVKVIAAHKEKGPTIGVDVYEGEPADML

ERGVIEPLRVKKQAIKSASEAAIMILRIDDVIAASKL

***Pyrococcus furiosus* chaperonin Pf-Cpn mutant CD1, namely the wild type without the last 22 amino acids, with Arg (R)instead of Ile or His at position 138 (Ile148Arg).**

**Name: Pf-CD1 Arg 138; Pf-R in short. (527 amino acids).**

MAQLAGQPILILPEGTQRYVGRDAQRMNILAARIVAETIRTTLGPKGMDKMLVDSLGDIVITNDGATILD

EMDIQHPAAKMMVEVAKTQDKEAGDGTTTAVVIAGELLRKAEELLDQNIHPSIIIKGYTLAAQKAQERLE

NIAKEVKPDDEEILLKAAMTSITGKAAEEEREYLAKLAVEAVKLVAEKEDGKYKVDIDNIKLEKKEGGSV

RDTQLIRGVVIDKEVVHPGMPKRVEKAKIALINDALEVKETETDAEIRITSPEQLQAFLEQEERMLREMV

EKIKEVGANVVFVQKGIDDLAQHYLAKYGIMAVRRVKKSDMEKLAKATGAKIVTNIRDLTPEDLGYAELV

EERKVAGESMIFVEGCQNPKAVTILIRGGTEHVVDEVERALEDAIKVVKDILEDGKILAGGGAPEIELAI

RLDEYAKEVGGKEQLAIEAFAEALKVIPRTLAENAGLDPIETLVKVIAAHKEKGPTIGVDVYEGEPADML

ERGVIEPLRVKKQAIKSASEAAIMILRIDDVIAASKL

**2.2. Protein production**

Recombinant BL21 (DE3) cells were inoculated into 15 mL of LB medium plus 50 μg/μL kanamycin at 37 °C, and this 15 mL culture was transferred into 1 L of ZYM Auto-Induction medium with kanamycin selection 10 h later. Cells were grown in 4 L baffled flasks at 200 rpm at 25 °C for 3 days. The cell pellets were re-suspended in re-suspension buffer (25 mM Tris-HCl, 200 mM NaCl and pH 7.5), 30 % w/v prior to cell disruption using a continuous flow Cell Disrupter (TS Series Benchtop, Constant Systems Ltd. GA, USA) and centrifuged at 11,500 x*g* for 20 min to collect cellular debris. The supernatant extract was heated to 80 °C (Pf-CD1 and Pf-H) or 70 °C (Pf-R) for 30 min and centrifuged at 11,500 x*g* for 30 min to remove non-heat tolerant proteins. The supernatants were further puriﬁed to homogeneity by anion exchange chromatography (Bio-Rad MiniMacro Prep High Q Column, Hercules, CA, USA), using a 200 mM to 1 M NaCl gradient for 30 min at 3 mL/min.

**2.3. Differential scanning calorimetry (DSC)**

The sample cell was filled with 0.3 mL of protein solution and the reference cell with the same volume of buffer. Measurements were carried out from 25 to 120 °C, at 60 °C/h scan rate, with various concentrations in PBS buffer (20 mM NaH_2_PO_4_, 100 mM NaCl, pH 7.5). Samples and buffers were ﬁltered and degassed in vacuum before loading into the DSC cell. Baselines were obtained by performing blank buffer-buffer scans.

The total enthalpy change needed for the complete unfolding, ∆H*_cal_*, was calculated by integrating the excess heat capacity of the unfolding transition, Δ*C_P,trs_*, over temperature in the significant temperature interval:

$${\Delta H}_{cal}= \int_{T_{1}}^{T_{2}} {\Delta C}_{P,trs}dT$$

*T*_1_ and *T*_2_ represent absolute temperature values before and after the unfolding process, respectively. Nano-Analyze TA and MicroCal Origin Pro 8.0 Software were used for baseline subtraction and ∆H*_cal_* calculation.

The van’t Hoff enthalpy (∆H*_vh_*), was calculated by assuming a simple two-state model. The calorimetric enthalpy is determined independently of any model. T_m_ indicates the apparent melting temperature.

**2.4. Isothermal titration calorimetry (ITC): oligomeric equilibrium**

Prior to use, protein solutions and buffer were filtered and degassed in vacuum. Each protein solution was loaded into the titration syringe at a concentration of 0.3 mg/mL. Each titration consisted of 18-19 successive injections of 5 µL protein solution into the reaction cell (980 µL) filled with buffer only. The time interval between consecutive injections was 5 min and stirring at 300 rpm was applied to ensure complete mixing. The heat values were obtained by integrating each thermal power peak. Data analysis and modeling utilized Nano-Analyze Data Analysis TA® and MicroCal Origin 7.0 software. All the equations and thermodynamic parameters are given in terms of moles of protein monomer (M). ITC curves were analysed by using a hexadecamer-monomer equilibrium model.

**2.5. Isothermal titration calorimetry: ATP binding**

Buffer: 20 mM Tris-HCl, 20 mM MgCl_2_ and 10 mM KCl (pH 7.6), kept at 37 °C. ATP concentration was determined by the absorbance at 259 nm using an extinction coefﬁcient of 15,400 M^-1^ cm^-1^. Prior to use, all solutions were degassed under vacuum. Titration experiments were performed by successive 5 μL injections of a freshly prepared 200 μM ATP solution into a protein solution (Pf-H and Pf-R, 0.42 μM for the hexadecamer). The time interval between consecutive injections was 300 s with stirring at 300 rpm. Binding isotherms were corrected by subtracting the ligand dilution isotherms, determined by titrating ATP solution into buffer.

For all three proteins, the dependence of the heat of reaction on the mole of titrant added conforms to a simple model of one set of ligand binding sites. The model assumes that there is a single independent binding site on each subunit forming 1:1 ligand/protein complex. The algorithm describing the data analysis for ATP binding is described in the Supplementary Material.

**ITC Analysis**

**Analytical Methods**

**Algorithm for the analysis of hexadecamer-monomer equilibrium by ITC**

The equilibrium constant and mass balance equations are in this case:

$$M_{16}\leftrightarrow16M$$

$$K_{d}=\frac{\left[ M \right]^{16}}{\left[ M_{16} \right]}$$

$$\left[ M \right]_{t}=\left[ M \right]+ 16\left[ M_{16} \right]$$

$$16\frac{\left[ M \right]^{16}}{K_{d}}+\left[ M \right]-\left[ M \right]_{t}=0$$

Here *K_d_* is the dissociation equilibrium constant, [*M*] and [*M_16_*] are the equilibrium concentrations of the monomer and hexadecamer, respectively, and [*M*]*_t_* is the total monomer concentration. The total concentration of protein (in a monomer base) in the calorimetric cell after each injection *i* can be calculated as:

$$\left[ M \right]_{t,i}=P_{0}\left( 1-\left( 1-\frac{v_{inj}}{V_{0}} \right)^{i} \right)$$

The free monomer concentration after each injection *i*, [*M*]*_i_*, can be calculated by using the previous equations, and the normalized heat associated with each injection, *Q_i_,* is given by:

$$Q_{i}=\frac{V_{0}\Delta H_{d}}{v_{inj}P_{0}}\left( \left[ M \right]_{i}-\left[ M \right]_{i-1}\left( 1-\frac{v_{inj}}{V_{0}} \right)-F_{0}P_{0}\frac{v_{inj}}{V_{0}} \right)+q_{d}$$

Where ∆*H_d_* is the enthalpy change due to dissociation, *V*_o_ is the reaction volume, *v*_inj_ the injected volume, *P_o_* is the molar concentration in the syringe, and *F_0_* is the fraction of monomers in the syringe.

The heat values were obtained by integrating each thermal power peak. Data analysis and modeling were carried out using Nano-Analyze Data Analysis TA® and MicroCal Origin 7.0 software. All the equations and thermodynamic parameters are given in terms of moles of protein monomer (M).

**Algorithm for the analysis of ATP binding by ITC**

In this case, the analytical solution for the normalized heat associated with each injection is:

$$Q_{i}=\frac{V_{0}\Delta H}{v_{inj}L_{0}}\left( \left[ PL \right]_{i}-\left[ PL \right]_{i-1}\left( 1-\frac{v_{inj}}{V_{0}} \right) \right)+q_{d}$$

where *V_0_* is the reaction volume, *∆H* is the enthalpy of binding, [*PL*]*_i_* is the concentration of protein-ligand complex in the reaction volume after each injection *i*, *v_inj_* is the injected volume, *L_0_* is the ligand concentration in the syringe, and *q_d_* is the background injection heat effect. The concentration of protein-ligand complex after each injection *i*, [*PL*]*_i_*, is calculated by solving and applying the following equations:

$${\left[ P \right]_{t,i}\frac{K\left[ L \right]_{i}}{1+K\left[ L \right]_{i}}+\left[ L \right]_{i}-[L]}_{t,i}=0$$

$$\left[ PL \right]_{i}=\left[ P \right]_{t,i}\frac{K\left[ L \right]_{i}}{1+K\left[ L \right]_{i}}$$

Where *K* is the equilibrium binding constant, and [*P*]*_i_* and [*L*]*_i_* are equilibrium concentrations on protein and ligand, respectively. The total concentrations of protein and ligand in the calorimetric cell after each injection *i* are given by:

$$\left[ P \right]_{t,i}=P_{0}\left( 1-\frac{v_{inj}}{V_{0}} \right)^{i}$$

$$\left[ L \right]_{t,i}=L_{0}\left( 1-\left( 1-\frac{v_{inj}}{V_{0}} \right)^{i} \right)$$

Data fit to the model provides the equilibrium constant *K* and the change of enthalpy *∆H* as fitting parameters. The corresponding changes of free energy, *∆G*, and entropy, *∆S,* are then determined by:

$$\Delta G=-RTlnK$$

$$\Delta G=\Delta H-T\Delta S$$

where *R* is the gas constant and *T* the absolute temperature.

**Supplementary Table S1**

Secondary structure composition and assignments from CD spectra for native and unfolded protein

| **Protein** | **Alpha-Helix^a^** | **Beta-Sheet** | **Turn** | **Random Coil** |
| --- | --- | --- | --- | --- |
| Pf-CD1 native | 50 | 12 | 12 | 26 |
| Pf-CD1 unfolded | 41 | 23 | 10 | 26 |
| Pf-H native | 49 | 21 | 10 | 20 |
| Pf-H unfolded | 40 | 24 | 10 | 26 |
| Pf-R native | 50 | 21 | 9 | 20 |
| Pf-R unfolded | 44 | 20 | 8 | 27 |

^a^Percent of alpha-helix, beta-sheet, turn and random coil structure obtained by CDPro analysis of CD spectra [24].

**
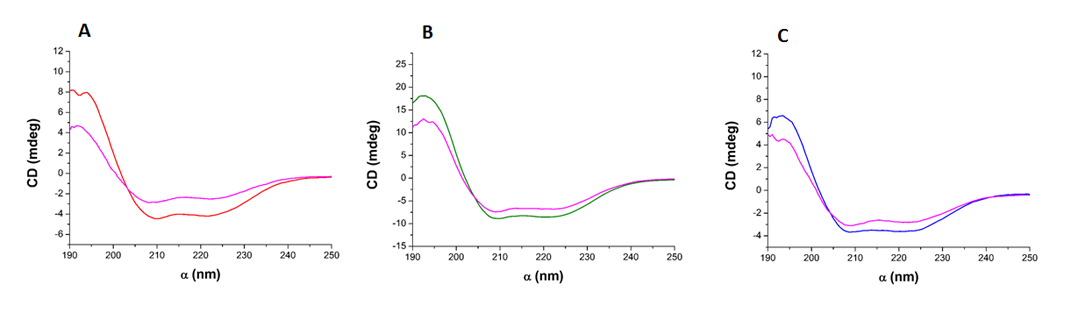
Supplementary Fig. S1.** Circular dichroism. CD spectra for Pf-CDl (panel A), Pf-H (panel B), and Pf-R (panel C) before (continuous lines) and after (dashed lines) thermal denaturation. All samples show a predominance of alpha helical structure (maxima near 190 nm and minima near 210 and 225nm), which is slightly reduced in unfolded samples. Secondary structure composition and assignments from CD spectra are given in Table S1.


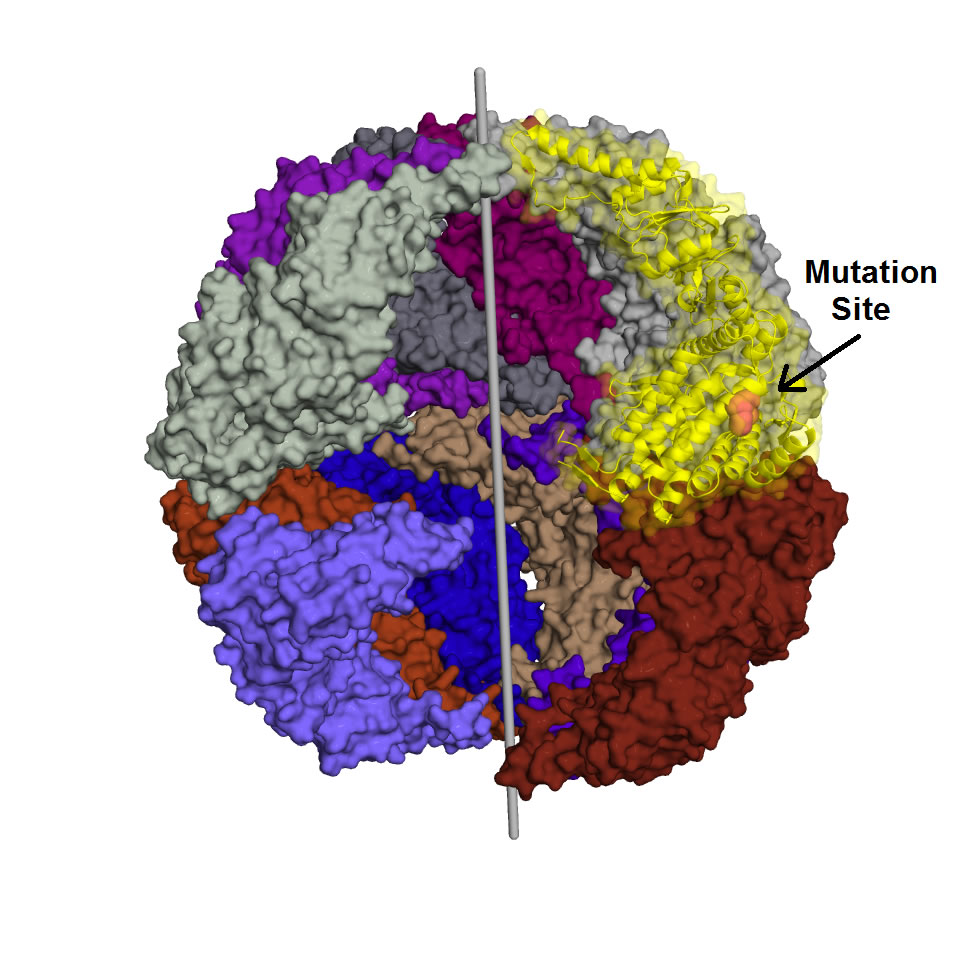


**Supplementary Fig. S2.** Cutaway view of the chaperonin hexadecamer, which is a hollow ball of radius 10 nm. The 8-fold symmetry axis is shown as a grey pole with subunits in various colors. Each hemisphere comprises 8 subunits, which although identical in archaea are here shown in different colors for the purpose of illustration. The four nearest subunits have been removed (two from the upper hemisphere and two from the lower) to show the interior chamber. The yellow subunit is rendered transparent to show the polypeptide fold. The site of the disease-causing mutation is indicated in one subunit only but it is present at the same locations in all other subunits of the *Pyrococcus furiosus* mutant used as experimental model in this study. Image made using the Pymol molecular graphics program, based on crystal structure 1Q3Q

**
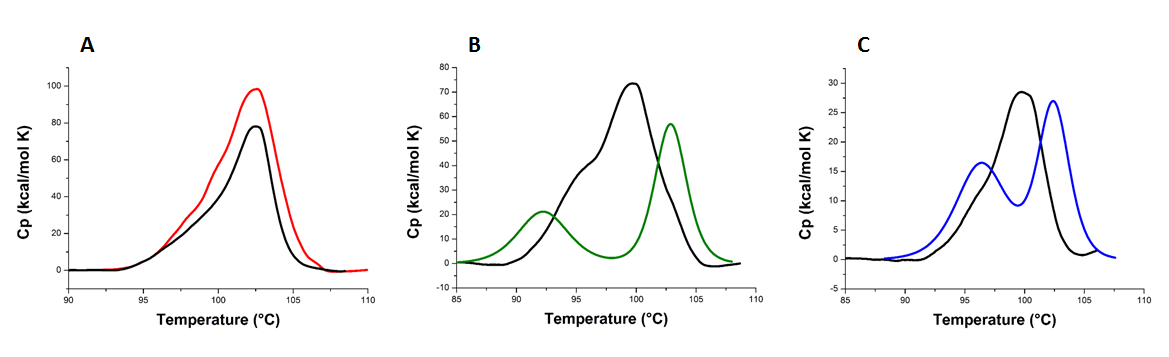
**

**Supplementary Fig. S3**. Differential scanning calorimetry proﬁles. The profiles show the dependence of the heat capacity on temperature in the absence of nucleotides, as follows: Panel A, Pf-CD1 red line; panel B, Pf-H, green line; and panel C, Pf-R blue line; and in the presence of 0.015 mM ATP (black line in the three panels).
