# Supplementary material for: Quantitative analysis of the impact of a human pathogenic mutation on the CCT5 chaperonin subunit using a proxy archaeal ortholog
Source: Biochem Biophys Rep. 2017 Sep 1;12:66–71. doi: 10.1016/j.bbrep.2017.07.011 (PMC5851525; doi:10.1016/j.bbrep.2017.07.011)
Supplement: Supplementary file 1 — Supplementary material [file mmc1.zip › Conflict of Interest.doc]

Conflict of Interest.
